# Mid-Regional Pro-Adrenomedullin, Methemoglobin and Carboxyhemoglobin as Prognosis Biomarkers in Critically Ill Patients with COVID-19: An Observational Prospective Study

**DOI:** 10.3390/v13122445

**Published:** 2021-12-06

**Authors:** Crhistian-Mario Oblitas, Francisco Galeano-Valle, Jesús Ramírez-Navarro, Jorge López-Cano, Ángel Monterrubio-Manrique, Mercedes García-Gámiz, Milagros Sancho-González, Sara Arenal-López, Luis-Antonio Álvarez-Sala Walther, Pablo Demelo-Rodríguez

**Affiliations:** 1 Internal Medicine Department, Hospital General Universitario Gregorio Marañón, 28007 Madrid, Spain; crhistian.cao@gmail.com (C.-M.O.); j.ramireznavarro7@gmail.com (J.R.-N.); lalvarezsalaw@gmail.com (L.-A.Á.-S.W.); pbdemelo@hotmail.com (P.D.-R.); 2 School of Medicine, Universidad Complutense de Madrid, 28040 Madrid, Spain; jorgelopezcano97@gmail.com (J.L.-C.); amonterr@ucm.es (Á.M.-M.); 3 Sanitary Research Institute Gregorio Marañón, 28007 Madrid, Spain; 4 Laboratory Medicine Department, Hospital General Universitario Gregorio Marañón, 28007 Madrid, Spain; mggamiz@salud.madrid.org; 5 Intensive Care Department, Hospital General Universitario Gregorio Marañón, 28007 Madrid, Spain; milagros.sancho@hotmail.com (M.S.-G.); sarenal.hgugm@salud.madrid.org (S.A.-L.)

**Keywords:** biomarkers, COHb, MetHb, mortality, MR-pro-ADM, SARS-CoV-2, thrombosis

## Abstract

Mid-regional pro-adrenomedullin (MR-proADM), methemoglobin (MetHb), and carboxyhemoglobin (COHb) levels have been associated with sepsis. In this study, we assessed the role of this potential biomarkers in critically ill COVID-19 patients. Outcomes were mortality and a combined event (mortality, venous or arterial thrombosis, and orotracheal intubation (OTI)) during a 30-day follow-up. A total of 95 consecutive patients were included, 51.6% required OTI, 12.6% patients died, 8.4% developed VTE, and 3.1% developed arterial thrombosis. MetHb and COHb levels were not associated with mortality nor combined event. Higher MR-proADM levels were found in patients with mortality (median of 1.21 [interquartile range-IQR-0.84;2.33] nmol/L vs. 0.76 [IQR 0.60;1.03] nmol/L, *p* = 0.011) and combined event (median of 0.91 [IQR 0.66;1.39] nmol/L vs. 0.70 [IQR 0.51;0.82] nmol/L, *p* < 0.001); the positive likelihood ratio (LR+) and negative likelihood ratio (LR−) for mortality were 2.40 and 0.46, respectively. The LR+ and LR− for combined event were 3.16 and 0.63, respectively. MR-proADM ≥1 nmol/L was the optimal cut-off for mortality and combined event prediction. The predictive capacity of MR-proADM showed an area under the ROC curve of 0.73 (95% CI, 0.62–0.81) and 0.72 (95% CI, 0.62–0.81) for mortality and combined event, respectively. In conclusion, elevated on-admission MR-proADM levels were associated with higher risk of 30-day mortality and 30-day poor outcomes in a cohort of critically ill patients with COVID-19.

## 1. Introduction

The coronavirus disease 2019 (COVID-19), caused by the severe acute respiratory syndrome coronavirus 2 (SARS-CoV2), is one of the main health issues over the world with more than 220 million confirmed cases and more than 4.5 million deaths. Approximately 20% of hospitalized patients with COVID-19 require admission to intensive care unit (ICU), with a mortality rate up to 40% [1,2].

The leading cause of mortality in patients with COVID-19 is hypoxemic respiratory failure from acute respiratory distress syndrome (ARDS). The pathophysiological mechanisms of SARS-CoV-2 infection are complex and poorly understood. In early stages, the virus infects the endothelial and epithelial cells of the lower respiratory tract triggering an inflammatory response. In late stages, this (disproportionate) inflammatory cascade will cause tissue damage and interstitial edema, compromising the integrity of the pulmonary interstitial barrier. Moreover, COVID-19 leads to a pro-coagulative state that increases the risk of thromboembolic phenomena (arterial and venous) [2,3]. In this setting of immune dysregulation, proinflammatory cytokines release may occur (misnamed “cytokine storm”) in response to systemic inflammatory response syndrome [4,5].

Adrenomedullin (ADM) is a biomarker that has been linked to endothelial dysfunction and the risk for organ failure in patients with sepsis and infection of the lung as it directly relates to the status of the endothelium. ADM shows a short half-life (approximately 22 min) and has a pro-peptide, the mid-regional proADM (MR-proADM), with a longer half-life and a 1:1 ratio in plasma, which makes it an ideal biomarker for the indirect quantification of ADM. [6,7,8,9,10].

Methemoglobin (MetHb) is a form of oxidized hemoglobin incapable of efficiently transporting oxygen, hence favoring hypoxemia. The presence of an excess of nitric oxide, together with anemia, seems to favor small sustained increases in MetHb in patients with infectious diseases or in severe patients, as an indicator of oxidative stress [11,12,13]. Along the same lines, a pro-inflammatory and pro-oxidative environment favors the overexpression of carboxyhemoglobin (COHb), favoring hypoxemia [14,15]. Both MetHb and COHb have been evaluated as biomarkers in patients with sepsis, septic shock, and in pediatric patients with severe malaria, suggesting that elevated levels have a prognostic value for short-term morbidity and mortality [12,13,14,15]. A recent systematic review of studies evaluating MetHb or COHb in COVID-19 patients only found four cross-sectional studies, concluding that their levels may be elevated, especially in critically ill patients [16].

The aim of this study was to assess the relationship between MR-proADM, MetHB, and COHb levels measured at ICU admission in critically ill patients with COVID-19 and early adverse outcomes.

## 2. Materials and Methods

### 2.1. Study Design and Patients

This was a single-center prospective observational study conducted between August and November 2020 that included all consecutive critically ill patients aged ≥ 18 diagnosed with SARS-CoV-2 infection admitted to the Intensive Care Unit of the Hospital General Universitario Gregorio Marañón, Spain (MR-proADM/MetHb/COHb_COVID).

The aim of the study was to assess the association between MR-proADM, MetHb, and COHb levels measured at ICU admission and mortality or a combined event (that included mortality, arterial thrombosis, venous thromboembolism, and the need of orotracheal intubation) during a 30-day follow-up. Local protocol for thromboprophylaxis consisted in enoxaparin 40 mg per day or bemiparin 3500 UI per day.

COVID-19 was diagnosed by a positive result of real-time reverse transcriptase-polymerase chain reaction testing or antigenic test of a nasopharyngeal specimen. COVID-19 ARDS was diagnosed on the basis of the Berlin 2012 ARDS diagnostic criteria: acute hypoxemic respiratory failure, presentation within one week of worsening respiratory symptoms; bilateral airspace disease on chest X-ray, computed tomography or ultrasound that is not fully explained by effusions, lobar or lung collapse or nodules; and cardiac failure is not the primary cause of acute hypoxemic respiratory failure [17].

Exclusion criteria were: (a) patients < 18 years; (b) pregnancy; (c) patients transferred from or to other hospital; (d) known history of cytochrome b5 reductase deficiency, hemoglobinopathies, glucose 6-phosphate dehydrogenase deficiency, or NADPH-MetHb reductase deficiency; (e) carbon monoxide poisoning.

This study was approved by the Ethics Committee of the Hospital General Universitario Gregorio Marañón and performed under a waiver of informed consent.

### 2.2. Measurement of MR-proADM Concentration by Quantitative Enzyme-Linked Immunosorbent Assay

A sample of blood from an EDTA-containing tube obtained at ICU admission was centrifugated at 4000 rpm for five minutes and then a plasma aliquot was immediately frozen and stored at −80 °C. When sufficient samples were collected to complete the capacity of the instrument, the MR-proADM measures were determined by the B.R.A.H.M.S. KRYPTOR compact PLUS (Thermo Fisher Scientific, Hennigsdorf, Germany) automated method using the TRACE (Time-Resolved Amplified Cryptate Emission) technique. Reference values were P95 0.52 nmol/L, median 0.39 nmol/L. The detection limit of the assay was 0.05 nmol/L. Reagents were supplied by ThermoScientific (BRAHMS Iberia S.L.).

### 2.3. Measurement of MetHb and COHb Concentration by Co-Oximetry

Blood gases were determined through a GEM Premier 4000 analyzer, Instrumentation Laboratory (Werfen) with Potentiometric sensors to measure pCO2, pH, Na+, K+, Cl−, and Ca++ and amperometric electrodes to measure pO2, glucose and lactate concentrations. MetHb and COHb obtained at ICU admission were estimated by spectrophotometric method (co-oximetry) by this analyzer.

### 2.4. Data Collection and Outcomes

Baseline characteristics, clinical history, laboratory findings and outcomes were collected from electronic medical records. Disease onset was defined as the day when the symptoms were noticed. SOFA (Sequential Organ Failure Assessment) [18] score and SEIMC (Spanish Society of Infectious Diseases and Clinical Microbiology) score [19] were calculated at ICU admission. SEIMC score classifies patients according the 30-day mortality risk into low (0–2 points), moderate (3–5 points), high (6–8 points), and very high risk (≥9 points). Off-label use of anti-inflammatory treatments for COVID-19 (i.e., steroid treatment, tocilizumab) was also recorded.

All patients were followed-up for 30 days or until hospital discharge. The primary adverse outcome was overall mortality. The secondary adverse outcome was a combined event that included overall mortality, arterial thrombosis, venous thromboembolism (VTE), and the need for orotracheal intubation (OTI).

### 2.5. Statistical Analysis

The Shapiro–Wilk test determined the normality of continuous quantitative variables. The study reported categorical data as proportions and continuous data as mean and standard deviation (SD) or median and inter-quartile range (IQR), depending on their normality. We used Student’s *t* test and analysis of variance (ANOVA) to compare means in 2 or more independent categories, respectively, when the variable followed a normal distribution. The nonparametric alternatives were the Mann–Whitney U and the Kruskal–Wallis test, respectively. Linear regression and Spearman’s rank correlation coefficient estimated the relationship between 2 continuous variables. The Kaplan–Meier estimator was used to graphically represent the events (death, VTE, arterial thrombosis, IOT, and combined event). The ROC (receiver operating characteristic) curve analysis determined the predictive capacity of MR-proADM, MetHb, and COHb and was used to determine a cut-off point for predicting adverse outcomes. The univariate logistic regression test determined the association between the cut-off point of MR-proADM, MetHb, and COHb levels and the adverse outcomes (crude odds ratio, OR) and multivariate logistic regression (MLR) test determined their age-adjusted association with a confidence interval (CI) at 95% level. All tests were two sided and the level of statistical significance was set at 0.05.

## 3. Results

### 3.1. Characteristic of Study Population

A total of 95 patients diagnosed with COVID-19 were admitted to the ICU during the study period, with a mean age of 60.3 ± 12.7 years (67.4% males). Patients’ characteristics are detailed in Table 1. All patients were treated with low molecular weight heparin at prophylactic doses and high-dose corticosteroids (dexamethasone ≥20 mg/day), 98.9% received antibiotic therapy (at least in monotherapy), 40% received tocilizumab (at least one dose), and 43.2% received remdesivir. Anakinra was used in 9% patients, lopinavir/ritonavir in 5%, colchicine in 2%, and hydroxychloroquine in 2%. Respiratory support was distributed as follows: invasive mechanical ventilation (IMV) in 51.6% patients, high flow oxygen therapy (Optiflow™) in 40%, Bilevel Positive Airway Pressure (BiPAP) in 2%, and continuous positive airway pressure (CPAP) in 2%.

Median duration of hospitalization was 27 (17–43) days and the median length of stay before ICU admission was 3 (1–6) days. During the study period, 12 patients died (12.6%) (11 died during ICU stay), among them 11 patients died due to ARDS and 1 patient died due to massive myocardial infraction at admission. Eight (8.4%) patients presented VTE, 3 (3.1%) patients presented arterial thrombosis, and 49 (51.6%) patients needed OTI. No patient was lost to follow up. The adverse outcomes are detailed in Table 2.

### 3.2. Characteristic of MetHb and COHb in COVID-19 Patients

No differences were found between median MetHb levels in patients who died (0.85% [0.7;1.3] vs. 1.1% [0.95;1.35]%, *p* = 0.340) and in patients with the combined event (1.0% [0.8;1.3] vs. 1.15% [1.0;1.4], *p* = 0.112). Likewise, no differences were found between median COHb levels in patients who died (1.7% [1.1;2.2] vs. 1.7% [1.3;1.9], *p* = 0.710) and in patients with the combined event (1.6% [1.1;2] vs. 1.7% [1.45;1.9], *p* = 0.522). The predictive capacity of MetHb showed a poor area under the ROC curve (AUC) for both mortality and combined event (0.40 and 0.59, respectively). Similarly, COHb showed a poor AUC for both mortality and combined event (0.53 and 0.53, respectively).

### 3.3. Characteristic of MR-proADM in COVID-19 Patients

**Figure 1:**
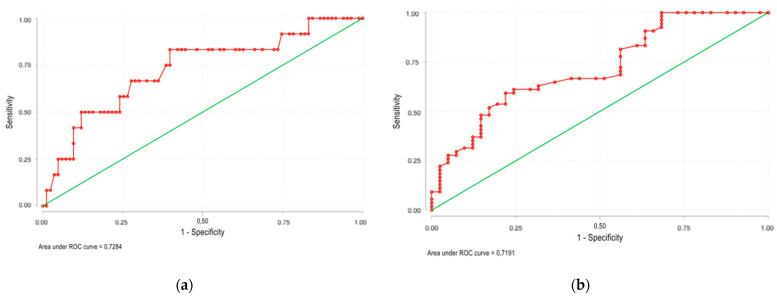
The predictive capacity of MR-proADM showed an area under the ROC curve of 0.73 (95% CI, 0.63–0.81; *p* = 0.017) for 30-day mortality (**a**) and 0.72 (95% CI, 0.62–0.81; *p* = 0.002) for 30-day combined event (**b**).

MR-proADM was measured only once within the first 72 h of ICU admission, with a median time elapsed of 42 h. Higher median MR-proADM levels were found in patients who died (1.21 [0.84;2.33]) nmol/L vs. 0.76 [0.60;1.03] nmol/L, *p* = 0.011) and in those with combined event (0.91 [0.66;1.39] nmol/L vs. 0.70 [0.51;0.82] nmol/L, *p* < 0.001). The predictive capacity of MR-proADM showed an AUC of 0.73 (95% CI, 0.63–0.81; *p* = 0.017) for 30-day mortality and 0.72 (95% CI, 0.62–0.81; *p* = 0.002) for 30-day combined event (Figure 1); the positive likelihood ratio (LR+) and negative likelihood ratio (LR−) for mortality were 2.40 and 0.46, respectively. The LR+ and LR− for combined event were 3.16 and 0.63, respectively. MR-proADM ≥ 1 mmol/L was the optimal cut-off point for both 30-day mortality prediction and 30-day combined event prediction. The negative predictive values were 93.8% and 54.7%, respectively.

**Figure 2:**
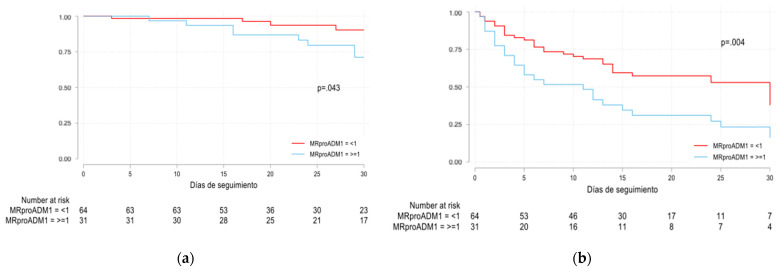
Overall survival Kaplan–Meier analyses of mortality (*p* = 0.043) (**a**) and combined event (*p* = 0.004) (**b**) stratified according to MR-proADM levels ≥ 1 mmol/L (blue) and <1 mmol/L (red). Absolute number of surviving patients (for mortality and combined event, respectively) on days 0, 5, 10, 15, 20, 25 and 30 comparing levels of MR-proADM < 1 mmol/L and ≥1 mmol/L, respectively.

Univariate logistic regression analysis showed that MR-proADM levels ≥1 mmol/L and age (years) were associated with mortality (crude OR 5.22 [95% CI 1.42–19.1; *p* = 0.013] and 1.14 [95% CI 1.03–1.26; *p* = 0.011], respectively). Similarly, MR-proADM levels ≥1 mmol/L and glomerular filtration rate (GFR) were associated with the combined event (crude OR 5.03 [95% IC 1.8–14; *p* = 0.002] and 0.89 [95% CI 0.83–0.96; *p* = 0.002], respectively). However, multivariate logistic regression analysis showed that MR-proADM levels ≥ 1 mmol/L were not independently associated with mortality nor combined event (adjusted OR 2.62 [95% CI 0.4–17.1; *p* = 0.314] and 3.04 [95% CI 0.9–10.8; *p* = 0.086], respectively) (Table 3). Univariate Cox regression analysis estimated a hazard ratio (HR) for MR-proADM levels ≥ 1 mmol/L of 3.23 (95% CI 0.97;10.73; *p* = 0.055) and 2.1 (95% CI 1.25;3.52; *p* = 0.005) for mortality and combined event, respectively (Figure 2).

Other scores were assessed. The predictive capacity of Sepsis-related Organ Failure Assessment (SOFA) score showed an AUC of 0.75 (95% CI, 0.64–0.86) for mortality and 0.79 (95% CI, 0.71–0.87) for 30-day combined event. However, there were no significant differences when combining the ROC curves of SOFA score and MR-proADM for mortality (*p* = 0.51) with an AUC of 0.79 (95% CI, 0.68–0.89) and 30-day combined event (*p* = 0.69) with an AUC of 0.8 (95% CI, 0.72–0.88) (Figure S1 in Supplementary Materials). Univariant and multivariant logistic regression for SOFA score are shown in Table 3.

On the other hand, five patients were classified as low risk, 29 patients as moderate risk, and 61 patients as high risk according SEIMC score and were associated with mortality (0%, 0%, and 80.3%, respectively; *p* = 0.008 for comparison of low-risk vs. high-risk and moderate risk vs. high risk). The SEIMC score ≥6 points showed an AUC of 0.83 (95% CI, 0.72–0.95) for mortality. The combination of MR-proADM levels ≥1 mmol/L and SEIMC score ≥6 points did not improve the prognostic capacity of the score for mortality (*p* = 0.771).

## 4. Discussion

The present study found no association between levels of MetHb and COHb and adverse outcomes, suggesting that both biomarkers may not be useful predictors. In this regard, a systematic review by Scholkmann et al. [20] found a possible relationship between a slight increase of MetHb and COHb with the severity of SARS-CoV2 infection. Paccaudi et al. [21] evaluated the potential association between COHb levels and SARS-CoV-2 infection severity with inconclusive results.

On the other hand, the present study showed that MR-proADM levels measured at ICU admission in COVID-19 patients are associated with 30-day mortality and combined event (overall mortality, arterial thrombosis, VTE, and the need for OTI); also, a cut-off point of 1 mmol/L showed a good prognostic capacity with a high negative predictive value (93.8%). However, SOFA score was the only factor independently associated with both mortality and the combined event in the MLR and the addition of MR-proADM did not improve the predictive capacity of SOFA. These results differ from those from other studies where MR-proADM was independent in the MLR showing prognostic capacity (Table S1 in Supplementary Materials). This difference might be related to the relatively low rate of events or the limited sample size. To our knowledge, this is the largest study evaluating the role of MR-proADM in critically ill COVID-19 patients. Other studies have evaluated the role of MR-proADM in the setting of COVID-19 pandemic [22,23,24,25,26,27,28,29,30]. Benedetti et al. [22] evaluated MR-proADM and other biomarkers in 21 ICU patients (measured on the 1st, 2nd, 3rd, and 5th day of admission) to determine their predictive value for mortality; the optimal cut-off for MR-proADM was 1.07 nmol/L (sensitivity of 91% and specificity of 71%; *p* = 0.006). Montrucchio et al. [23] evaluated MR-proADM (measured on the 2nd, 3rd, 7th, and 14th days of admission) in 57 patients admitted to the ICU to assess mortality. The optimal cut-off was 1.8 nmol/L, showing an adjusted OR of 10.3 (95% CI, 1.9–53.6; *p* = 0.006), and an AUC of 0.85 (95% CI, 0.78–0.90). Roedl et al. [24] evaluated the predictive value of MR-proADM and the requirement for renal replacement therapy (RRT) for extrarenal clearance in 64 patients at ICU admission. MR-proADM showed an AUC of 0.69 (95% CI, 0.54–0.83) to predict the need for RRT, with an adjusted OR of 3.8 (95% CI, 1.1–13.1). Spoto et al. [25] evaluated the capacity of MR-proADM to predict the development of ARDS and mortality in 69 hospitalized patients. The optimal cut-off for mortality was 2 nmol/L with an AUC of 0.89 (S 78.6% and E 88%), and a HR of 12.3 (95% CI, 2.66–57.28). Gregoriano et al. [26] evaluated MR-proADM at hospital admission in 89 patients with COVID-19. The optimal cut-off point for predicting mortality was 0.93 nmol/L. Zaninotto et al. [29] retrospectively evaluated the potential role of MR-proADM in detecting endothelial dysfunction and its usefulness for stratifying COVID-19 patients; with a median time elapsed from the hospital admission to MR-proADM measurement of 7 days. A total of 135 patients were included, and the findings suggested that higher MR-proADM levels are associated with a greater risk for adverse outcomes. Table 4 summarizes the studies evaluating MR-proADM in COVID-19 patients; it should be noted that these studies have been carried out during the first wave, when patients were more likely to present higher severity and mortality. On the other hand, patients from our study were enrolled during the second wave and therefore were more likely to be younger, with fewer comorbidities and lower mortality [31,32].

Our study has some strengths: the role of MR-proADM was analyzed in terms of clinical practice, and attempts were made to avoid information biases when establishing the definitions of the concepts in the methods. Selection biases were avoided by including all consecutive patients admitted to intensive care during the study period; also, there were no losses to follow-up. On the other hand, the present work has some limitations: it is an observational study and the variables recorded were collected in a healthcare context; some possible confounding factors were not analyzed in the multivariate analysis (clinical variables or the treatment received). As it is a single-center study, its results cannot be extrapolated to other populations. Likewise, it was performed in an intensive care unit, so its results might not be applied to non-critical patients; and MR-proADM was measured only once and the dynamic changes of the biomarkers were not assessed. Future studies including patients from multi-centers and in both critically and non-critically COVID-19 patients are needed to ascertain these findings.

## 5. Conclusions

The medial region of proadrenomedullin (MR-proADM), but not carboxyhemoglobin or methemoglobin, is a useful biomarker to predict mortality and development of complications at 30 days in critically ill patients with SARS-CoV-2 infection. More studies are needed to validate these results in other populations, including non-critical patients.

## Figures and Tables

**Table 1:** Baseline characteristics and laboratory findings at ICU admission.

Variable		Total (*n* = 95)	Survivors (*n* = 83)	Non-Survivors (*n* = 12)	*p* Value
Sex male, *n* (%)		64 (67.4)	55 (66.3)	9 (75)	0.75
BMI, *n* (%)	<25	23 (24.2)	20 (24.1)	3 (25)	1.00
	25–30	40 (42.1)	35 (42.2)	5 (41.7)	1.00
	≥30	32 (33.7)	28 (33.7)	4 (33.3)	1.00
Age, years (mean, SD)		60.3 ± 12.8	58.7 ± 12.5	71.3 ± 9.1	0.001
BMI, kg/m^2^ (mean, SD)		29 ± 5	29 ± 4.7	29 ± 6.8	0.86
Hemoglobin, mg/dL (median, P25–P75)		13.3 (12–14.6)	13.3 (12–14.6)	13.3 (12.3–13.9)	0.98
Anemia (hemoglobin <12 g/dl), *n* (%)		26 (27.4)	23 (27.7)	3 (25)	1.00
Leukocytes, µL^−1^ (median, P25–P75)		10,400 (7500–12,800)	10,200 (7500–12,800)	11,555 (9200–13,900)	0.23
Neutrophils, µL^−1^ (median, P25–P75)		9300 (6200–11,700)	9000 (6100–11,400)	10,850 (8150–12,850)	0.12
Lymphocytes, µL^−1^ (median, P25–P75)		600 (400–1000)	700 (400–1000)	450 (300–650)	0.04
Lymphocytes <1000/µL, *n* (%)		68 (71.6)	57 (68.7)	11 (91.7)	0.17
Neutrophil/lymphocyte ratio, (median, P25–P75)		13.6 (7.3–23)	13.4 (6.8–22.2)	30.9 (12.8–42.3)	0.02
Neutrophil/lymphocyte ratio, *n* (%)	<3.22	5 (5.3)	5 (6)	0 (0)	1.00
	3.22–6.53	13 (13.7)	12 (14.5)	1 (8.3)	1.00
	>6.53	77 (81.1)	66 (79.5)	11 (91.7)	0.45
Platelets, ×1000∙µL^−1^ (median, P25–P75)		241 (194–288)	242 (196–292)	212 (147–249)	0.17
Platelets ≤ 150,000/µL, *n* (%)		14 (14.7)	10 (12)	4 (33.3)	0.07
INR ≥ 1.25, *n* (%)		15 (15.8)	12 (14.5)	3 (25)	0.4
D-Dimer, ng/mL (median, P25–P75)		577 (331–1061)	563 (307–820)	1124 (438–2710)	0.046
D-Dimer, *n* (%)	≥600 ng/mL	45 (47.4)	38 (45.8)	7 (58.3)	0.54
	≥1000 ng/mL	24 (25.3)	17 (20.5)	7 (58.3)	0.01
Ferritin ≥ 274 µg/L, *n* (fraction)		67/78 (86)	61/69 (88.4)	6/9 (66.7)	0.11
IL-6 ≥ 4.3 pg/mL, *n* (fraction)		76/80 (95)	65/69 (94.2)	11/11 (100)	1.00
LDH ≥ 225 U/L, *n* (%)		91 (95.8)	79 (95.2)	12 (100)	1.00
Glomerular filtration rate, *n* (%)	<60 mL/min/1.73 m^2^	14 (14.7)	10 (12)	4 (33.3)	0.07
	<30 mL/min/1.73 m^2^	6 (6.3)	4 (4.8)	2 (16.7)	0.16
Total bilirubin ≥1.2 mg/dL, *n* (%)		6 (6.3)	5 (6)	1 (8.3)	0.57
C-reactive protein, mg/dL (median, P25–P75)		13.6 (6.3–24.6)	13.3 (6.3–23.8)	19.2 (6.4–27.1)	0.52
C-reactive protein, *n* (%)	≥1 mg/dL	90 (94.7)	79 (95.2)	11 (91.7)	0.5
	≥5 mg/dL	75 (78.9)	65 (78.3)	10 (83.3)	1.00
	≥8 mg/dL	65 (68.4)	57 (68.7)	8 (66.7)	1.00
Procalcitonin, µg/L (median, P25–P75)		0.13 (0.05–0.73)	0.12 (0.05–0.46)	0.5 (0.05–2.66)	0.19
Procalcitonin ≥0.5 µg/L, *n* (%)		25 (26.3)	19 (22.9)	6 (50)	0.07
MR-proADM, nmol/L (median, P25–P75)		0.77 (0.61–1.14)	0.76 (0.6–1.03)	1.22 (0.84–2.33)	0.01
MR-proADM, *n* (%)	≥0.75 nmol/L	53 (55.8)	43 (51.8)	10 (83.3)	0.06
	≥1 nmol/L	29 (30.5)	23 (27.7)	8 (66.7)	0.02
MetHb, %Hb total (mean, SD)		1.09 ± 0.39	1.1 ± 0.38	1.03 ± 0.56	0.34
MetHb ≥ 1%, *n* (fraction)		25/86 (29.1)	57/76 (75)	4/10 (40)	0.06
COHb, %Hb total (mean, SD)		1.57 ± 0.52	1.57 ± 0.5	1.62 ± 0.73	0.71
COHb > 1.3%, *n* (fraction)		20/86 (23.3)	60/76 (78.9)	6/10 (60)	0.23
Arterial pH ≤ 7.35, *n* (%)		16 (16.8)	11 (13.3)	5 (41.7)	0.03
Arterial lactate ≥ 0.8 mmol/L, *n* (%)		89 (93.7)	78 (94)	11 (91.7)	0.57
Duration of symptoms before admission, days (median, P25–75)		6 (3–8)	6 (4–8)	2 (1–6)	0.02
Length of stay, days (median, P25–P75)		12 (6–30)	12 (6–32)	14 (4–23)	0.34
SOFA score, median (P25–75)		2 (2–4)	2 (2–4)	4 (3–6.5)	0.003
SOFA score, *n* (%)	1	2 (2.1)	2 (2.4)	0 (0)	1.00
	2	47 (49.5)	47 (56.6)	0 (0)	0.0002
	3	10 (10.5)	5 (6)	5 (41.7)	0.003
	4	13 (13.7)	11 (13.3)	2 (16.7)	0.67
	5	6 (6.3)	4 (4.8)	2 (16.7)	0.16
	≥6	17 (17.9)	14 (16.9)	3 (25)	0.45
SEIMC score, *n* (%)	3–5 Moderate	5 (5.3)	5 (6)	0 (0)	1.00
	6–8 High	29 (30.5)	29 (34.9)	0 (0)	0.02
	≥9 Very high	61 (64.2)	49 (59)	12 (100)	0.004

COHb: carboxyhemoglobin; BMI: body mass index; INR: international normalized ratio; MetHb: methemoglobin; MR-proADM: mid-regional pro-adrenomedullin; SEIMC: Spanish Society of Infectious Diseases and Clinical Microbiology; SOFA: Sequential (sepsis-related) Organ Failure Assessment.

**Table 2:** Events during 30-day follow-up.

Variable (*n* = 95)		*n* (%)
Mortality, *n* (%)		12 (12.6)
Time until death, days (median, P25–75)		18.5 (13.5–25.5)
Place of death, *n* (%)	ICU	11/12 (87.5)
	After ICU discharge	1/12 (12.5)
Cause of death, *n* (%)	COVID-19	11/12 (87.5)
	Acute myocardial infarction	1/12 (12.5)
VTE, *n* (%)		8 (8.4)
Time until VTE, days (median, P25–P75)		14 (9.5–17)
Arterial thrombosis, *n* (%)		3 (3.1)
Time until arterial thrombosis, days (median, P25–P75)		7 (2–17)
OTI, *n* (%)		49 (51.6)
Time until OTI, days (median, P25–P75)		1 (0–2)
Combined event *, *n* (%)		54 (56.8)

ICU: intensive care unit. OTI: orotracheal intubation; VTE: venous thromboembolism. * Combined event includes the patients with ≥1 of the following: VTE, arterial thrombosis, OTI, or death.

**Table 3:** Univariate and multivariate logistic regression analysis for 30-day mortality and 30-day combined event.

Variables	OR	95% CI		*p* Value	OR	95% CI		*p* Value
	Mortality				Combined Event			
	**Univariate Logistic Regression Analysis**							
Age (years)	1.14	1.03	1.26	0.011	1.02	0.99	1.05	0.244
Oxygen saturation (%)	0.96	0.90	1.03	0.296	0.98	0.92	1.05	0.554
Neutrophils/lymphocytes ratio	1.03	0.99	1.05	0.07	1.01	0.98	1.05	0.375
Glomerular filtration rate (mL/min·1.73 m^2^)	0.96	0.92	1.004	0.077	0.89	0.83	0.96	0.002
Sex (male)	0.65	0.16	2.63	0.551	0.89	0.37	2.11	0.785
Procalcitonin ≥ 1 ng/mL	2.69	0.70	10.34	0.149	2.06	0.66	6.43	0.215
C-reactive protein ≥ 8 mg/dl	1.09	0.30	3.99	0.889	0.81	0.34	1.95	0.641
MR-proADM ≥ 1 mmol/L	5.22	1.42	19.14	0.013	5.03	1.81	13.99	0.002
COHb ≥ 1.3%	2.50	0.62	10.02	0.196	2.66	0.86	8.21	0.09
MetHb ≥ 1%	3.75	0.96	14.68	0.058	2.92	0.95	8.96	0.062
SOFAscore	1.28	1.08	1.52	0.005	2.4	1.52	3.78	0.000
	**Multivariate Logistic Regression Analysis**							
Age (years)	1.17	1.03	1.32	0.014	1.03	0.99	1.07	0.19
Glomerular filtration rate (ml/min·1.73 m^2^)	0.97	0.92	1.03	0.34	0.96	0.91	1.02	0.18
MR-proADM ≥ 1 mmol/L	1.29	0.17	9.48	0.8	1.73	0.46	6.49	0.42
SOFAscore	1.38	1.01	1.89	0.04	2.23	1.44	3.45	0.000

CI: confidence interval; OR: odds ratio; SOFA: Sepsis-related Organ Failure Assessment. * Multivariate logistic regression analysis was performed for variables that presented *p* value <0.1 in the univariate analysis.

**Table 4:** Studies evaluating MR-proADM levels as prognostic for 30-day mortality in patients with COVID-19.

Author	*n*	Age	% ICU Patients	SOFA Score	MR-proADM Levels (nmol/L)			*n* (%) Deaths	Cut-Off Point for Death (nmol/L)	AUC for 30 Day Mortality
					Total Sample	Survivors	Non-Survivors			
Benedetti I et al. [22]	21	70.9 (54–85)	23.8%	3.5 ±2.3	2.3 ±2.7	1.1 (mean)	2.3 (mean)	11 (52.4%)	1.07	0.81
Montrucchio G et al. [23]	57	64 (54–71)	100%	7 (4–10)	2 ± 1.3	1.22 ±0.49	2.74 ±1.99	31 (54.4%)	1.8	0.85 (95%CI 0.78–0.9)
Spoto S et al. [25]	69	78 (61–84)	43.5%	2 (1–7)	1.49 (0.67–2.26)	1.15 (0.57–1.85)	5.25 (2.67–6.53)	16 (23.2%)	2.00	0.89
Gregoriano C et al. [26]	89	67 (58–74)	26%	NR	NR	0.8 (0.7–0.11)	1.3 (1.1–2.3)	17 (19.1%)	0.93	0.78
García de Guadiana-Romualdo L et al. [27]	99	66 ±15	16.2%	NR	0.74 (0.6–1.02)	0.68 (0.57–0.94)	1.54 (1.05–2.12)	14 (14.1%)	0.88	0.91 (95% CI 0.82–0.95)
Sozio E et al. [28] ^‡^	111	62.3 ± 13.6	25.2% *	2 (1–3)	0.82 (0.64–1.08)	0.73 (0.56–0.94) **	1.38 (0.94–1.73) **	28 (25.2%) **	0.9 **	0.85 (95% CI 0.77–0.73) **
Zaninotto M et al. [29] ^‡^	135	67 (58–77)	52.6%	NR	0.93 (0.64–1.46)	NR	NR	14 (10.4%)	0.5–1.5 ^‡‡^	0.9 (95% CI 0.827–0.974)
Lo Sasso B et al. [30] ^‡^	110	62 (52–76)	1.82%	NR	0.93 (0.58–1.09)	0.82 (0.57–1.03)	2.59 (2.3–2.95)	14 (12.7%)	1.73	0.95 (95% CI 0.86–0.99
Present study	95	60.3 ± 12.7	100%	2 (2–4)	0.77 (0.61–1.14)	0.76 (0.60–1.03)	1.21 (0.84–2.33)	12 (12.6%)	1	0.73 (95% CI 0.63–0.81)

Variables are expressed as mean ± SD or median (P25-P75) as shown in the articles. * In this study, the percentage of patients admitted to the ICU was not available. Therefore, it has been assumed equal to the number of patients who required orotracheal intubation. ** The objective of this study was the predictive value of MR-proADM for a combined event (death and need for orotracheal intubation), therefore data regarding mortality alone were not available. ^‡^ Retrospective studies. ^‡‡^ The samples were stratified by 3 groups: group 1 (*n* = 20, MR- proADM ≤ 0.55 nmol/L), group 2 (*n* = 82, 0.55 nmol/L < MR-proADM ≤ 1.50 nmol/L), and group 3 (*n* = 33, MR-proADM > 1.50 nmol/L). AUC: Area Under the ROC Curve; ICU: intensive care unit; MR-proADM: mid-regional pro-adrenomedullin; NR: not registered; SOFA: Sequential (sepsis-related) Organ Failure Assessment; CI: confidence interval.

## Data Availability

All the data are present in the main text.

## Supplementary Materials

The following are available online at https://www.mdpi.com/article/10.3390/v13122445/s1, Figure S1: ROC curve for SOFA score, Table S1: Studies evaluating the prognostic capacity of MR-proADM levels in patients with COVID-19.

## References

[1] Coronavirus COVID-19 Global Cases by the Center for Systems Science and Engineering (CSSE) at Johns Hopkins University. https://coronavirus.jhu.edu/.

[2] Wiersinga W.J., Rhodes A., Cheng A.C., Peacock S.J., Prescott H.C. (2020). Pathophysiology, transmission, diagnosis, and treatment of Coronavirus disease 2019 (COVID-19): A review: A review. JAMA.

[3] Guan W.J., Ni Z.Y., Hu Y., Liang W.H., Ou C.Q., He J.X. (2020). China Medical Treatment Expert Group for Covid-19. Clinical Characteristics of Coronavirus Disease 2019 in China. N. Engl. J. Med..

[4] Sinha P., Matthay M.A., Calfee C.S. (2020). Is a “Cytokine Storm” Relevant to COVID-19?. JAMA Intern Med..

[5] Kox M., Waalders N.J.B., Kooistra E.J., Gerretsen J., Pickkers P. (2020). Cytokine Levels in Critically Ill Patients with COVID-19 and Other Conditions. JAMA.

[6] Mearelli F., Barbati G., Casarsa C., Giansante C., Breglia A., Spica A., Moras C., Olivieri G., Occhipinti A.A., De Nardo M. (2020). The Integration of qSOFA with Clinical Variables and Serum Biomarkers Improves the Prognostic Value of qSOFA Alone in Patients with Suspected or Confirmed Sepsis at ED Admission. J. Clin. Med..

[7] Valenzuela-Sánchez F., Valenzuela-Méndez B., Rodríguez-Gutiérrez J.F., Estella A., González-García M. (2016). New role of biomarkers: Mid-regional pro-adrenomedullin, the biomarker of organ failure. Ann. Transl. Med..

[8] Buendgens L., Yagmur E., Ginsberg A., Weiskirchen R., Wirtz T., Abu Jhaisha S., Eisert A., Luedde T., Trautwein C., Tacke F. (2020). Midregional Proadrenomedullin (MRproADM) Serum Levels in Critically Ill Patients Are Associated with Short-Term and Overall Mortality during a Two-Year Follow-Up. Mediat. Inflamm..

[9] Elke G., Bloos F., Wilson D.C., Brunkhorst F.M., Briegel J., Reinhart K. (2018). SepNet Critical Care Trials Group. The use of mid-regional proadrenomedullin to identify disease severity and treatment response to sepsis–A secondary analysis of a large randomised controlled trial. Crit. Care.

[10] Andaluz-Ojeda D., Nguyen H.B., Meunier-Beillard N., Cicuéndez R., Quenot J.-P., Calvo D., Dargent A., Zarca E., Andrés C., Nogales L. (2017). Superior accuracy of mid-regional proadrenomedullin for mortality prediction in sepsis with varying levels of illness severity. Ann. Intensiv. Care.

[11] Gordo-Remartínez S., Calderón-Moreno M., Fernández-Herranz J., Castuera-Gil A., Gallego-Alonso-Colmenares M., Puertas-López C., Nuevo-González J.A., Sánchez-Sendín D., García-Gámiz M., Sevillano-Fernández J.A. (2015). Usefulness of midregional proadrenomedullin to predict poor outcome in patients with community acquired pneumonia. PLoS ONE.

[12] Hare G.M., Tsui A.K., Crawford J.H., Patel R.P. (2013). Is methemoglobin an inert bystander, biomarker or a mediator of oxidative stress--The example of anemia?. Redox Biol..

[13] Ohashi K., Yukioka H., Hayashi M., Asada A. (1998). Elevated methemoglobin in patients with sepsis. Acta Anaesthesiol. Scand..

[14] Behera G.C., Behera S.K., Jena R.K., Bharati V.S. (2015). Study of Methaemoglobin in Malaria Patients. Indian J. Hematol. Blood Transfus..

[15] Yeo T.W., Lampah D.A., Kenangalem E., Tjitra E., Price R.N., Anstey N.M. (2013). Increased carboxyhemoglobin in adult falciparum malaria is associated with disease severity and mortality. J. Infect. Dis..

[16] Hänscheid T., Gresnigt T., Löhr S., Flamen A., Zoller T., Melo-Cristino J., Grobusch M.P. (2014). Methaemoglobin and COHb in patients with malaria. Malar. J..

[17] Ranieri V.M., Rubenfeld G.D., Thompson B.T., Ferguson N.D., Caldwell E., Fan E., Camporota L., Slutsky A.S., ARDS Definition Task Force (2012). Acute Respiratory Distress Syndrome: The Berlin Definition. JAMA.

[18] Vincent J.L., Moreno R., Takala J. (1996). Working Group on Sepsis-Related Problems of the European Society of Intensive Care Medicine. The SOFA (Sepsis-related Organ Failure Assessment) score to describe organ dysfunction/failure. Intensive Care Med..

[19] Berenguer J., Borobia A.M., Ryan P., Rodríguez-Baño J., Bellón J.M., Jarrín I., Carratalà J., Pachón J., Carcas A.J., Yllescas M. (2021). Development and validation of a prediction model for 30-day mortality in hospitalised patients with COVID-19: The COVID-19 SEIMC score. Thorax.

[20] Scholkmann F., Restin T., Ferrari M., Quaresima V. (2020). The Role of Methemoglobin and Carboxyhemoglobin in COVID-19: A Review. J. Clin. Med..

[21] Paccaud P., Castanares-Zapatero D., Gerard L., Montiel V., Wittebole X., Collienne C., Laterre P.F., Hantson P. (2020). Arterial carboxyhemoglobin levels in Covid-19 critically Ill patients. Res. Sq..

[22] Benedetti I., Spinelli D., Callegari T., Bonometti R., Molinaro E., Novara E., Cassinari M., Frino C. (2021). High levels of mid-regional proadrenomedullin in ARDS COVID-19 patients: The experience of a single, Italian Center. Eur. Rev. Med. Pharmacol. Sci..

[23] Montrucchio G., Sales G., Rumbolo F., Palmesino F., Fanelli V., Urbino R. (2021). Effectiveness of mid-regional pro-adrenomedullin (MR-proADM) as prognostic marker in COVID-19 critically ill patients: An observational prospective study. PLoS ONE.

[24] Roedl K., Jarczak D., Fischer M., Haddad M., Boenisch O., de Heer G., Burdelski C., Frings D., Sensen B., Karakas M. (2021). MR-proAdrenomedullin as a predictor of renal replacement therapy in a cohort of critically ill patients with COVID-19. Biomarkers.

[25] Spoto S., Agrò F.E., Sambuco F., Travaglino F., Valeriani E., Fogolari M., Mangiacapra F., Costantino S., Ciccozzi M., Angeletti S. (2021). High value of mid-regional proadrenomedullin in COVID-19: A marker of widespread endothelial damage, disease severity, and mortality. J. Med. Virol..

[26] Gregoriano C., Koch D., Kutz A., Haubitz S., Conen A., Bernasconi L., Hammerer-Lercher A., Saeed K., Mueller B., Schuetz P. (2020). The vasoactive peptide MR-pro-adrenomedullin in COVID-19 patients: An observational study. Clin. Chem. Lab. Med..

[27] García de Guadiana-Romualdo L., Calvo Nieves M.D., Rodríguez Mulero M.D., Calcerrada Alises I., Hernández Olivo M., Trapiello Fernández W., Morales M.G., Jiménez C.B., Albaladejo-Otón M.D., Ovalle H.F. (2021). MR-proADM as marker of endotheliitis predicts COVID-19 severity. Eur. J. Clin. Investig..

[28] Sozio E., Tascini C., Fabris M., D’Aurizio F., De Carlo C., Graziano E., Bassi F., Sbrana F., Ripoli A., Pagotto A. (2021). MR-proADM as prognostic factor of outcome in COVID-19 patients. Sci. Rep..

[29] Zaninotto M., Maria Mion M., Marchioro L., Padoan A., Plebani M. (2021). Endothelial dysfunction and Mid-Regional proAdrenomedullin: What role in SARS-CoV-2 infected Patients?. Clin. Chim. Acta.

[30] Lo Sasso B., Gambino C.M., Scichilone N., Giglio R.V., Bivona G., Scazzone C., Muratore R., Milano S., Barbagallo M., Agnello L. (2021). Clinical Utility of Midregional Proadrenomedullin in Patients with COVID-19. Lab. Med..

[31] Saito S., Asai Y., Matsunaga N., Hayakawa K., Terada M., Ohtsu H., Tsuzuki S., Ohmagari N. (2020). First and second COVID-19 waves in Japan: A comparison of disease severity and characteristics. J. Infect..

[32] Bongiovanni M., Arienti R., Bini F., Bodini B.D., Corbetta E., Gianturco L. (2021). Differences between the waves in Northern Italy: How the characteristics and the outcome of COVID-19 infected patients admitted to the emergency room have changed. J. Infect..

