# Supplementary material for: Mid-Regional Pro-Adrenomedullin, Methemoglobin and Carboxyhemoglobin as Prognosis Biomarkers in Critically Ill Patients with COVID-19: An Observational Prospective Study
Source: Viruses. 2021 Dec 6;13(12):2445. doi: 10.3390/v13122445 (PMC8709066; doi:10.3390/v13122445)
Supplement: Supplementary file 1 [file viruses-13-02445-s001.zip › viruses-1433109-supplementary.pdf]

Supplementary appendix for Figures and Tables

- SOFA score Exitus

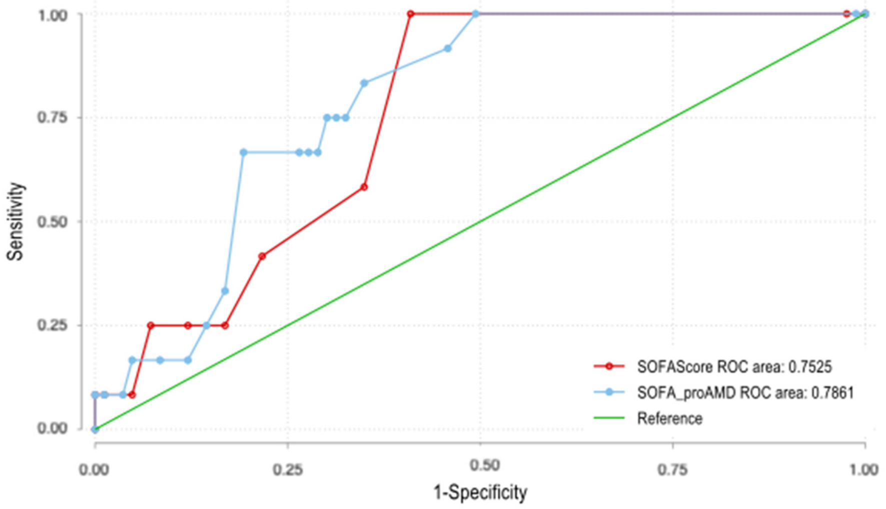

(a)

- SOFA score EndPoint

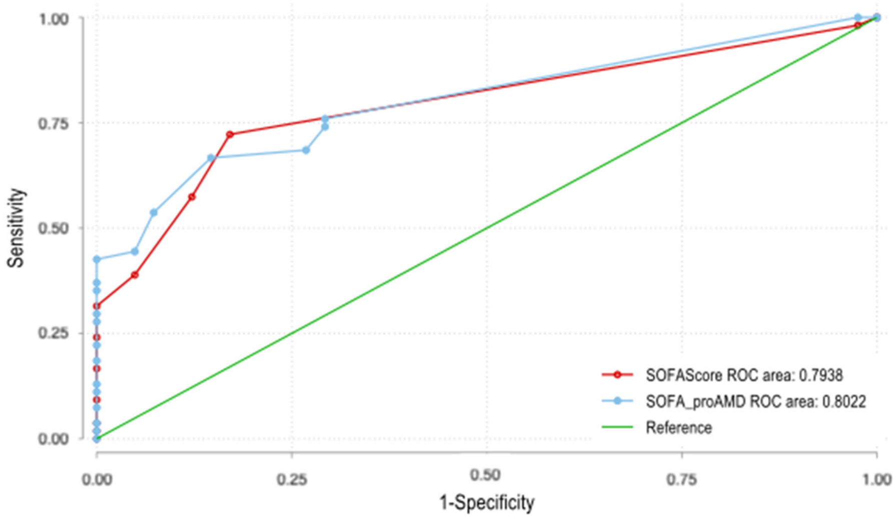

(b)

Figure S1. (a) The predictive capacity of SOFA score for 30-day mortality showed an area under the ROC curve of 0.75 (95% CI, 0.64-0.86) and AUC of 0.79 (95% CI, 0.68-0.89) when combined with MR-proADM (p=0.51 for the comparison); **(b)** The SOFA score AUC for 30-day combined event was 0.79 (95% CI, 0.71-0.88) and 0.8 (95% CI, 0.72-0.88) when combined with MR-proADM (p=0.69 for the comparison).

Table S1. Studies evaluating the prognostic capacity of MR-proADM levels in patients with COVID-19.

Author	n	OR of ULR (95% CI)	*p* value	OR of MLR (95% CI)	*p* value	Endpoint
Benedetti I et al (22)	21	ND	-	ND	-	
Montrucchio G et al (23)	57	ND	-	10.3 (1.9-56.3)	0.006	30-day Mortality
Spoto S et al (25)	69	ND	-	ND	-	
Gregoriano C et al (26)	89	3.2 (1.3-8.1)	0.012	5.5 (1.4-21.4)	0.015	30-day Mortality
García de Guadiana-Romualdo L et al (27)*	99	23.25 (5.19-104.15)	0.001	10.47 (2.07-53.05)	0.005	28-day Mortality
Sozio E et al (28)	111	4.33 (1.92-12.47)	0.002	4.28 (1.89-11.41)	0.0006	30-day Death or OTI
Zaninotto M et al (29)	135	2.48	0.000	ND	-	30-day Mortality
Lo Sasso B et al (30)	110	ND	-	ND	-	
Present study	95	5.22 (1.22-19.14)	0.013	1.29 (0.17-9.48)	0.8	30-day Mortality

*Data related to Hazard ratio Uni- and multivariate Cox regression analysis for 28-day mortality.
ND: no data; HR: hazard ratio; OR: odds ratio; OIT: orotracheal intubation; ULR: univariate logistic regression; MLR: multivariate logistic regression.
